# A 24-year pharmacovigilance study on sex differences in adverse drug reactions to antidepressant drugs

**DOI:** 10.1007/s00210-025-04900-7

**Published:** 2025-12-13

**Authors:** Johanna Seifert, Renate Grohmann, Sermin Toto, Matthias A. Reinhard, Stefan Bleich, Andreas Erfurth, Cornelius Schüle, Susanne Stübner, Catherine Glocker

**Affiliations:** 1 Department of Psychiatry, Social Psychiatry and Psychotherapy, Hannover Medical School, Carl-Neuberg-Straße 1, 30625 Hannover, Germany; 2 Department of Psychiatry and Psychotherapy, LMU University Hospital, Munich, Germany; 3 Department of Psychiatry and Psychotherapeutic Medicine, Klinik Hietzing, Vienna, Austria; 4 Department of Psychiatry and Psychotherapy, Medical University of Vienna, Vienna, Austria; 5 Department of Forensic Psychiatry and Psychotherapy, Psychiatric University Hospital Zurich, Rheinau, Switzerland

**Keywords:** Hyponatremia, Sexual dysfunction, Weight gain, Sex factors, Mental health

## Abstract

Depression is more prevalent in women. Antidepressant drugs (ADDs) are often associated with an increased risk for adverse drug reactions (ADRs). Women generally appear to be at greater risk for ADRs. This observational study analyzed the incidence of ADD-related ADRs in relation to sex in 151,426 female and 92,162 male psychiatric inpatients using data from the German pharmacovigilance program “Arzneimittelsicherheit in der Psychiatrie” (AMSP) from 1993 to 2016. The type of ADR and the incidence of ADRs for different ADDs are examined. Among women and men, 0.85% and 0.67% suffered from ADD-induced ADRs (relative risk 1.25, 95% confidence interval 1.15–1.35), respectively. Women had a higher incidence of edema (f 0.055% vs m 0.009%; RR 6.31, CI 3.06–13.04), allergic cutaneous reactions (f 0.057% vs m 0.034%; RR 1.71, CI 1.13–2.57), and hyponatremia (f 0.067% vs m 0.024%; RR 2.82, CI 1.78–4.47). The incidence of sexual dysfunction was significantly higher in men (m 0.044% vs f 0.001%; RR 17.95, CI 4.39–73.48). Almost all ADD classes had a higher incidence of ADRs in women, including selective serotonin reuptake inhibitors (f 0.61% vs m 0.46%; RR 1.25, CI 1.04–1.50), noradrenergic and specific serotonergic antidepressants (f 0.71% vs m 0.48%; RR 1.46, CI 1.18–1.81), and tricyclic antidepressants (f 1.02% vs m 0.92%; RR 1.24, CI 1.03–1.49). These findings highlight the importance of considering sex-specific tolerability of ADDs. An understanding of these differences is a prerequisite for the individualization and optimization of patient care.

**Supplementary Information:**

The online version contains supplementary material available at 10.1007/s00210-025-04900-7.

## Introduction

Depression, characterized by the two cardinal symptoms low mood and anhedonia alongside a host of additional symptoms, is a debilitating mental disorder that imposes a substantial burden on the affected individuals, their families, and the economy (König et al. 2019). Its incidence has increased by 50% within the past three decades (Liu et al. 2020). While the lifetime prevalence of depression varies by geographical region, epidemiological studies have consistently revealed that depression is twice as prevalent in women than in men (Li et al. 2023). The validity of this gender difference remains debated, with some researchers arguing that the 2:1 ratio is an artefact rooted in a lower treatment use and recognition of depression in men rather than an actual difference in prevalence (Kuehner 2017). Supporting this notion is the consideration that depression in men may present differently and with symptoms not captured in current diagnostic criteria, including substance misuse, poor impulse control, irritability, and aggressiveness (Möller-Leimkühler et al. 2004; Cavanagh et al. 2017). Currently, only second to low back pain, depressive disorders are among the top diseases for which women bear a higher burden than men, with an additional 348.3 disability-adjusted life years (DALYs) per 100,000 individuals in females compared to males (Patwardhan et al. 2024). Whatever the underlying cause, this disparity likely contributes to the twofold higher prescription rate of antidepressant drugs (ADDs) among women (Zhong et al. 2014; Lockhart and Guthrie 2011). This is compounded by the observation that female sex correlates with double the risk of adverse drug reactions (ADRs) (Zucker and Prendergast 2020), a wide range of which can occur during treatment with ADDs.

An ADR is defined as any unintended and harmful response to a drug during its use at normal doses for treatment, prevention, or diagnosis which requires further attention, such as specific treatment or alteration of the drug or dosing regimen (Edwards and Aronson 2000). Apart from their direct negative impact on health and patient well-being, ADRs are associated with a host of other complications including poorer treatment outcomes (Braund et al. 2021) and non-adherence to treatment (Uher et al. 2009). In fact, ADRs are one of the most commonly reported reasons for self-discontinuation of psychotropic drugs among patients with mental disorders (Samples and Mojtabai 2015; Velligan et al. 2017).

Women have historically found less consideration in clinical studies (Liu and Mager 2016), limiting our understanding of sex-related differences in ADRs and emphasizing the relevance of pharmacovigilance. Previous research has suggested a higher prevalence of adverse effects of ADD treatment such as nausea, dizziness, tremor, constipation, and weight gain in women (Haack et al. 2009; Bet et al. 2013). Men, on the other hand, are more likely to report and suffer from sexual dysfunction (Bet et al. 2013). However, although many of these ADRs are certainly bothersome, it is also important to consider clinical severity, which unfortunately is often not disclosed. A recent 2023 systematic review on sex differences in ADRs in relation to different drug classes was unable to find any relevant studies for ADDs (Shan et al. 2023), further emphasizing the current research gap.

The present study aims to provide a direct comparison of the incidence of severe ADD-induced ADRs in relation to sex by considering not only the various kinds of ADD-induced ADRs (e.g., hyponatremia, delirium, weight gain), but also the different classes of ADDs associated with ADRs. Further, it will consider ADRs occurring in relation to a single ADD and those implicating more than one drug. This differential approach is unique to the methods of the present study and is a reflection of real-life treatment practice. To the best of our knowledge, the present study is the first to comprehensively assess this matter within a large pharmacovigilance program.

## Methods

### The AMSP program and data collection

“Arzneimittelsicherheit in der Psychiatrie” (AMSP; German for “Drug Safety in Psychiatry”) was founded in 1993 and continues its work as an ongoing pharmacovigilance program in Germany and Austria. AMSP’s methodology has been described extensively elsewhere (Grohmann et al. 2004). In summary, AMSP gathers data on (1) general psychotropic drug use in hospitalized psychiatric patients and (2) ADRs related to psychotropic drug use, which is stored in two distinct databases. Data on drug use is gathered on two index days each year and comprises drug use and dosage, age, and sex of all patients that are currently in inpatient treatment in participating hospitals on the day of data collection. The number of patients treated in each hospital per year and the average duration of inpatient stay then allow an estimation of the number of patients exposed to each drug or combination of drugs. Reports on ADRs are collected in a standardized manner as they occur during inpatient treatment. All events of severe ADRs are included in the database, where they are categorized by the affected organ systems (e.g., neurological, cardiovascular). The severity of the ADRs is assessed according to AMSP standards as defined in Grohmann et al. (2004, 2014): An ADR is considered severe, “if it is (potentially) life-threatening or seriously endangers the patient’s health, if it considerably impairs everyday functioning or requires the patient’s transfer” for more specialized care. All AMSP data are collected and stored in an anonymized manner. The Ethics Committee of the University of Munich and Hannover Medical School (Nr. 8100_BO_S_2018) has approved analyses using the AMSP database. This study adheres to the Declaration of Helsinki and its later amendments. Due to its observational nature, AMSP does not interfere with the ongoing treatment of the patients under surveillance.

### Assessment and collection of adverse drug reactions

Physicians trained as drug monitors regularly screen inpatients treated in their hospital for any events of ADRs. These are then registered in detail using standardized questionnaires and carefully reviewed by experienced physicians. A plausibility rating for the relationship between the ADR and the implicated drug(s) is performed—i.e., “possible,” “probable,” “definite,” or “questionable”—according to AMSP standards (Grohmann et al. 2004). The present study only includes ADRs rated “probable” or “definite.” Only ADRs occurring in at least 15 cases are presented. ADRs are categorized according to the affected organ system (e.g., cardiovascular system, cutaneous reactions). Of note, AMSP designates certain ADR categories separately due to their high relevance in patients treated with psychotropic drugs. In so, the ADRs “delirium and confusion” are classified independently from other psychiatric ADRs and “EPS” are distinguished from “neurological ADRs.”

As a result of polypharmacy, in many cases, more than one drug is implicated to have caused the ADR in question via pharmacokinetic and/or pharmacodynamic interactions. The evaluation of causality of each drug is performed separately. Based on the number of drugs implicated, the present study distinguishes between three different groups of ADRs. The first group includes ADRs implicating only one drug (i.e., “single imputation”), the second includes ADRs implicating a combination of drugs (i.e., “multiple imputation”), and the third comprises both single and multiple imputations (i.e., “all cases”) (Grohmann et al. 2004).

In addition to the course of the ADR (e.g., fatal outcome, permanent damage) and its management (e.g., discontinuation of the causative drug, symptomatic treatment with drugs), clinical risk factors for ADRs are also reported. The present study only reports risk factors applicable in 100 or more ADR cases, which include “pre-existing organ damage,” which entails that the organ system affected by the ADR was already impaired beforehand (e.g., pre-existing liver damage), and “susceptibility for ADRs,” which refers in particular to the patient having previously developed ADRs to other drugs.

### Classification of psychotropic drugs relevant to the present study

Only ADDs used in ≥ 4000 patients were included in the further analysis of ADRs. ADDs were categorized as follows:

- *Selective serotonin reuptake inhibitors (SSRIs)*: escitalopram, citalopram, fluoxetine, fluvoxamine, paroxetine, sertraline
- *Selective serotonin-norepinephrine reuptake inhibitors (SSNRIs)*: duloxetine, venlafaxine, milnacipran
- *Tricyclic antidepressants (TCAs)*: amitriptyline, clomipramine, doxepin, imipramine, maprotiline, trimipramine
- *Noradrenergic and specific serotonergic antidepressants (NaSSAs)*: mianserin, mirtazapine
- *Monoamine oxidase inhibitors (MAOIs)*: moclobemide, tranylcypromine
- *Other ADDs*: agomelatine, bupropion, reboxetine, trazodone

### Statistical methods

Primary objectives of the present study were to determine (a) the incidence and relative risk (RR) of different types of ADRs and (b) the incidence and RR for ADRs for different ADDs according to sex. The incidence of ADRs is presented as percent of patients with an ADR/number of patients exposed to the respective drug/drug class. To determine the risk of different types of ADRs according to sex, we calculated the RR of different types of ADRs including the 95% confidence interval (CI). The risk of ADRs associated with different types of drugs was also calculated as RRs. RRs of > 1 suggest an increased ADR-risk for women than men, whereas RRs of < 1 signify a lower risk for women. Of note, the RRs reported here are derived from the observed incidence of ADRs and therefore represent estimates from which the actual risk can only be extrapolated.

Descriptive statistical analysis was performed using Excel^©^ and SPSS^©^ version 26 by IBM. General characteristics (i.e., age group, diagnosis) in women vs men were compared using chi-squared tests. In order to determine differences in the number of drugs used in women vs men, we first performed a Shapiro–Wilk test to assess normality. As the data was not normally distributed, a Welch’s *t*-test was then applied. We then calculated Cohen’s *d* as a measure of effect size (negligible *d* < 0.02, small *d* = 0.2, medium *d* = 0.5, large *d* = 0.8). The level of significance was set at *p* < 0.05.

## Results

### Characteristics of the study population

#### Characteristics according to sex

Between 1993 and 2016, AMSP monitored a total of 462,661 inpatients who were treated with one or more psychotropic drugs, 243,588 (52.6%) of whom were treated with at least one ADD. Among patients treated with ADDs, 151,426 (62.2%) were female and 92,162 (37.8%) were male. The percentage of females ≥ 65 years of age was significantly higher than that of males (*p* < 0.001). Depressive disorders were by far the most common diagnosis in both sexes; however, they were significantly more frequent in women. Neurotic, stress-related, somatoform, and personality disorders were also more common in women, while men were more often diagnosed with organic disorders, substance-related disorders, mania, and schizophrenia (*p* < 0.001; Table 1).

**Table 1:** Characteristics (i.e., age group, diagnosis) of the study population according to sex

	**All patients**		**Females**		**Males**		***χ***^**2**^** test (f vs m); df = 1**		**Post hoc *χ***^**2**^** test (f vs m); df = 6**	
	**All patients treated w/ADDs**	**% of all patients exposed to ADDs**	***N* females exposed ADDs**	**% of females exposed to ADDs**	***N* males exposed to ADDs**	**% of males exposed to ADDs**	***χ***^**2**^	***p*-value**	***χ***^**2**^	***p*-value**
**Total**	243,588	100%	151,426	100%	92,162	100%				
**Age group**										
** < 65 years**	187,010	76.8%	111,289	73.5%	75,721	82.2%	2412.81	< 0.001		
** ≥ 65 years**	56,578	23.2%	40,137	26.5%	16,441	17.8%				
**Diagnosis (ICD-10)**										
** Organic disorders (F0)**	17,769	7.3%	9695	6.4%	8074	8.8%	9859.71	< 0.001	470.78	< 0.001
** Substance-related disorders (F1)**	10,402	4.3%	4776	3.2%	5626	6.1%			1219.3	< 0.001
** Schizophrenia (F2)**	23,395	9.6%	9695	6.4%	13,700	14.9%			4725.05	< 0.001
** Depressive disorders (F3 without F30, F31.0–F31.2)**	142,216	58.4%	92,114	60.8%	50,102	54.4%			986.22	< 0.001
** Mania (F30, F31.0–F31.2)**	2035	0.8%	1089	0.7%	946	1.0%			64.93	< 0.001
** Neurotic/personality disorders (F40–48, F60–62)**	36,465	15.0%	24,077	15.9%	12,388	13.4%			271.88	< 0.001
** Others (F5, F63–F9)**	11,306	4.6%	9980	6.6%	1326	1.4%			3434.57	< 0.001

*N*, number (of); *ADD*, antidepressant drug; *w/*, with; *RR*, relative risk; *CI*, confidence interval; *f*, females; *m*, males; *ICD-10*, International Classification of Disease, 10th Version

#### Relative risk for adverse drug reactions according to age group and diagnosis

Overall, 0.83% of women and 0.67% of men treated with ADDs experienced an ADR (RR 1.25, CI 1.15–1.35). Of females < 65 years of age, 0.77% suffered from an ADD-associated ADR (vs 0.64% of men; RR 1.20, CI 1.10–1.32). Among females ≥ 65 years of age, 1.01% suffered from an ADD-associated ADR (vs 0.80% of men; RR 1.26, CI 1.06–1.50). Women with schizophrenia and depressive disorders also had a higher risk for ADD-associated ADRs compared to men with these diagnoses (Table 2).

**Table 2:** Relative risk for adverse drug reactions (ADRs) females and males according to age group and diagnosis

		**Females**			**Males**		**RR (f vs m)**
	***N* cases of ADD-induced ADRs**	***N* females exposed ADDs**	**% of females w/ADD-induced ADR**	***N* cases of ADD-induced ADRs**	***N* males exposed to ADDs**	**% of males w/ADD-induced ADRs**	**RR (95% CI)**
**Total**	1263	151,426	**0.83%**	616	92,162	**0.67%**	**1.25 (1.15–1.35)**
**Age group**							
< 65 yrs	857	111,289	**0.77%**	484	75,721	**0.64%**	**1.20 (1.10–1.32)**
≥ 65 yrs	406	40,137	**1.01%**	132	16,441	**0.80%**	**1.26 (1.06–1.50)**
**Diagnosis (ICD-10)**							
** Organic disorders (F0)**	59	9695	**0.61%**	41	8074	**0.51%**	**1.20 (0.88–1.63)**
** Substance-related disorders (F1)**	26	4776	**0.54%**	21	5626	**0.37%**	**1.46 (0.95–2.24)**
** Schizophrenia (F2)**	102	9695	**1.05%**	59	13,700	**0.43%**	**2.44 (1.89–3.16)**
** Depressive disorders (F3 without F30, F31.0–F31.2)**	919	92,114	**1.00%**	417	50,102	**0.83%**	**1.20 (1.09–1.32)**
** Mania (F30, F31.0–F31.2)**	15	1089	**1.38%**	6	946	**0.63%**	**2.17 (0.97–4.87)**
** Neurotic/personality disorders (F40–48, F60–62)**	136	24,077	**0.56%**	68	12,388	**0.55%**	**1.03 (0.81–1.31)**
** Others (F5, F63–F9)**	6	9980	**0.06%**	4	1326	**0.30%**	**0.20 (0.07–0.53)**

*N*, number (of); *ADD*, antidepressant drug; *w/*, with; *RR*, relative risk; *CI*, confidence interval; *f*, females; *m*, males; *yrs*, years; *ICD-10*, International Classification of Disease, 10th Version

#### Average number of different drug groups

Among patients with ADRs, the average number of drugs (f 4.05 ± 2.31 vs m 3.77 ± 2.29), psychotropic drugs (f 2.73 ± 1.33 vs m 2.71 ± 1.37), and ADDs (f 1.27 ± 0.49 vs m 1.29 ± 0.50) differed in only a clinically insignificant manner (i.e., Cohen’s *d* < 0.2) between women and men with ADRs. When comparing women with vs without ADRs, the average number of drugs (f with ADRs 4.05 ± 2.31 vs f without ADRs 4.08 ± 2.39), psychotropic drugs (f with ADRs 2.73 ± 1.33 vs f without ADRs 2.60 ± 1.32), and ADDs (f with ADRs 1.27 ± 0.49 vs f without ADRs 1.24 ± 0.46) did not significantly differ (Cohen’s *d* < 0.2). The same held true for the average number of drugs (m with ADRs 3.77 ± 2.29 vs m without ADRs 3.69 ± 2.25), psychotropic drugs (m with ADRs 2.71 ± 1.37 vs m without ADRs 2.51 ± 1.30), and ADDs (m with ADRs 1.29 ± 0.50 vs m without ADRs 1.23 ± 0.46) in men with vs without ADRs (Cohen’s *d* < 0.02 in all cases).

### Type of antidepressant drug-induced adverse drug reactions according to sex

The present study includes 1879 severe ADRs associated with ADDs. Overall, 1263 of these ADRs affected females, of which 1210 were “probable” imputations and 53 were “definite.” Among the 616 ADRs that affected males, 594 were “probable” and 22 were “definite” imputations.

#### All imputations (i.e., single and multiple imputation)

The most common type of ADD-associated ADRs were neurological ADRs (excl. EPS; 341 cases), and there within seizures (68 cases) and serotonin syndrome/serotonergic ADRs (52 cases). The second most common ADR type was cutaneous reactions (224 cases), especially allergic reactions (118 cases). Liver dysfunction followed in third place (176 cases).

The occurrence of the ADR delirium and confusion, psychiatric ADRs, extrapyramidal symptoms (EPS), gastrointestinal ADRs, liver dysfunction, cardiovascular ADRs, and urological ADRs did not show any sex-related differences. In contrast, women had a higher incidence of neurological ADRs (excl. EPS; f 0.155% vs m 0.116%; RR 1.33, CI 1.06–1.67). Among individual ADRs in this category, a significant difference was only detected for tremor, for which women had a higher incidence (f 0.019% vs m 0.009%; RR 2.21, CI 1.01–4.83). Women had a significantly higher incidence of adverse cutaneous reactions than men (f 0.121% vs m 0.044%; RR 2.72, CI 1.94–3.81), which was also detected for both of the two most relevant types of ADRs in this category, i.e., edema (f 0.055% vs m 0.009%; RR 6.31, CI 3.06–13.04) and allergic cutaneous reactions (f 0.057% vs m 0.034%; RR 1.71, CI 1.13–2.57). Similarly, metabolic disorders and electrolyte disorders occurred more commonly in women (f 0.077% vs m 0.027%; RR 2.82, CI 1.83–4.35), especially hyponatremia (f 0.067% vs m 0.024%; RR 2.82, CI 1.78–4.47) and symptomatic hyperprolactinemia/galactorrhea (f 0.009% vs m 0.001%; RR 8.52, CI 1.12–64.80). The incidence of sexual dysfunction was significantly lower among women (f 0.001% vs m 0.064%; RR 0.02, CI 0.01–0.08), corresponding to an 18-fold higher risk (RR 17.95, CI 4.39–73.48) in men compared to women, most commonly presenting as erectile dysfunction. Finally, the incidence of hematologic ADRs was higher among women than men (f 0.027% vs m 0.013%; RR 2.08, CI 1.09–3.96).

**Fig. 1:**
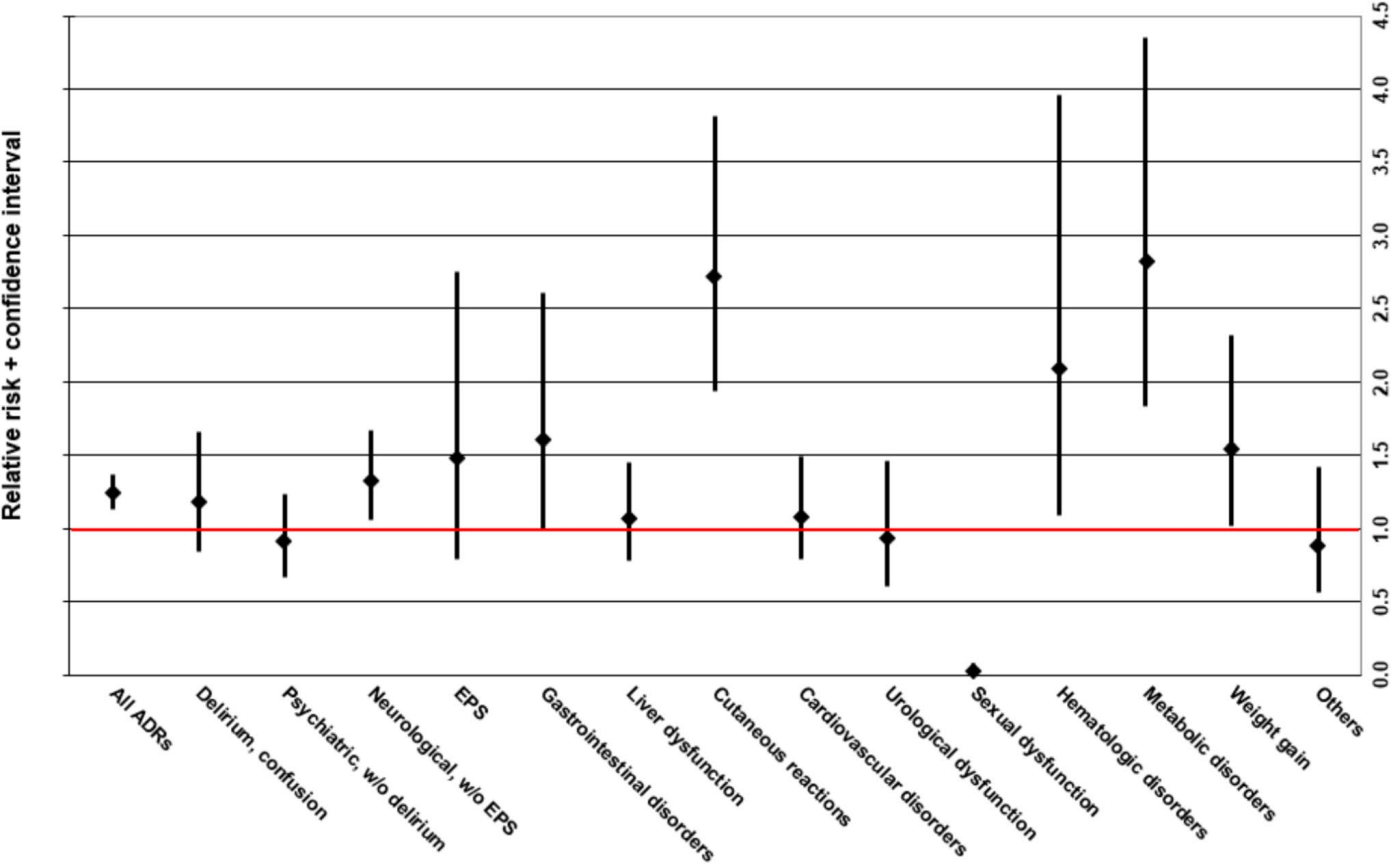
Relative risk (RR) incl. 95% confidence interval of adverse drug reactions (ADRs) affecting different organ systems (all imputations) in females vs males. RR > 1 implies a higher ADR-risk for females compared to males; RR < 1 implies a lower ADR-risk for females compared to males. w/, with; w/o, without; EPS, extrapyramidal symptoms

The sex-specific RR of all ADRs (i.e., single and multiple imputations) according to the affected organ system is depicted in Fig. 1. The exact number of ADR events and percent of patients affected, as well as the exact RRs including the 95% CIs of the affected organ system and of individual ADRs (cut-off ≥ 15 cases in all patients), are shown in Table 3.

**Table 3:** Incidence and relative risk of different types of adverse drug reactions (ADRs) associated with antidepressant drugs (ADDs; all imputations) according to sex

**Adverse drug reaction (cut-off 15 ADRs total)**	**Females (*N* = 151,426)**		**Males (*N* = 92,162)**		**RR (95% CI)**
	**All events of the ADR**		**All events of the ADR**		
	**N cases**	**% of females**	***N* cases**	**% of males**	
**All ADRs**	1263	**0.834%**	616	**0.668%**	1.25 (1.13–1.37)*
**Delirium, confusion**	97	**0.064%**	50	**0.054%**	**1.18 (0.84–1.66)**
**Psychiatric symptoms w/o delirium**	100	**0.066%**	67	**0.073%**	**0.91 (0.67–1.24)**
Psychosis/(pseudo-) hallucinations	14	**0.009%**	8	**0.009%**	**1.07 (0.45–2.54)**
Restlessness/agitation	33	**0.022%**	26	**0.028%**	**0.77 (0.46–1.29)**
Sedation	9	**0.006%**	6	**0.007%**	**0.91 (0.32–2.56)**
Suicidality	11	**0.007%**	9	**0.010%**	**0.74 (0.31–1.80)**
Nightmares	16	**0.011%**	7	**0.008%**	**1.39 (0.57–3.38)**
**Neurological symptoms w/o EPS**	234	**0.155%**	107	**0.116%**	1.33 (1.06–1.67)*
Seizures	43	**0.028%**	25	**0.027%**	**1.05 (0.64–1.71)**
Myoclonus	16	**0.011%**	5	**0.005%**	**1.95 (0.71–5.32)**
Ataxia	10	**0.007%**	5	**0.005%**	**1.22 (0.42–3.56)**
Tremor	29	**0.019%**	8	**0.009%**	2.21 (1.01–4.83)*
Vision disorders	12	**0.008%**	3	**0.003%**	**2.43 (0.69–8.63)**
Serotonin syndrome, serotonergic ADRs	36	**0.024%**	16	**0.017%**	**1.37 (0.76–2.47)**
Restless legs/arms	30	**0.020%**	19	**0.021%**	**0.96 (0.54–1.71)**
Vertigo	7	**0.005%**	9	**0.010%**	**0.47 (0.18–1.27)**
Speech disorders	7	**0.005%**	10	**0.011%**	**0.43 (0.16–1.12)**
**EPS**	34	**0.022%**	14	**0.015%**	**1.48 (0.79–2.75)**
**Gastrointestinal disorders**	61	**0.040%**	23	**0.025%**	**1.61 (1.00–2.61)**
(Sub)ileus/severe constipation	16	**0.011%**	7	**0.008%**	**1.39 (0.57–3.38)**
Nausea/vomiting	21	**0.014%**	6	**0.007%**	**2.13 (0.86–5.28)**
Diarrhea	14	**0.009%**	7	**0.008%**	**1.22 (0.49–3.02)**
**Liver dysfunction**	112	**0.074%**	64	**0.069%**	**1.07 (0.78–1.45)**
**Cutaneous reactions**	183	**0.121%**	41	**0.044%**	2.72 (1.94–3.81)*
Edema	83	**0.055%**	8	**0.009%**	6.31 (3.06–13.04)*
Allergic cutaneous reactions	87	**0.057%**	31	**0.034%**	1.71 (1.13–2.57)*
**Cardiovascular disorders**	105	**0.069%**	59	**0.064%**	**1.08 (0.79–1.49)**
(Orthostatic) syncope	43	**0.028%**	23	**0.025%**	**1.14 (0.69–1.89)**
Symptomatic hypotension with vertigo	13	**0.009%**	8	**0.009%**	**0.99 (0.41–2.39)**
Hypertension	15	**0.010%**	7	**0.008%**	**1.30 (0.53–3.20)**
Arrhythmia	25	**0.017%**	17	**0.018%**	**0.90 (0.48–1.66)**
**Urological dysfunction**	51	**0.034%**	33	**0.036%**	**0.94 (0.61–1.46)**
Urinary retention	48	**0.032%**	29	**0.031%**	**1.01 (0.64–1.60)**
**Sexual dysfunction**	2	**0.001%**	59	**0.064%**	0.02 (0.01–0.08)*
Erectile dysfunction, impotence	0	**0.000%**	41	**0.044%**	**–**
**Hematologic disorders**	41	**0.027%**	12	**0.013%**	2.08 (1.09–3.96)*
Neutropenia	12	**0.008%**	4	**0.004%**	**1.83 (0.59–5.66)**
Abnormal bleeding, bleeding disorder	16	**0.011%**	4	**0.004%**	**2.43 (0.81–7.28)**
**Metabolic disorders, electrolyte imbalances**	116	**0.077%**	25	**0.027%**	2.82 (1.83–4.35)*
Hyponatremia	102	**0.067%**	22	**0.024%**	2.82 (1.78–4.47)*
Symptomatic hyperprolactinemia/galactorrhea	14	**0.009%**	1	**0.001%**	8.52 (1.12–64.80)*
**Weight gain**	79	**0.052%**	32	**0.035%**	**1.50 (1.00–2.27)**
**Others**	44	**0.029%**	30	**0.033%**	**0.89 (0.56–1.42)**
Falls	9	**0.006%**	6	**0.007%**	**0.91 (0.32–2.56)**
Hyperhidrosis	19	**0.013%**	11	**0.012%**	**1.05 (0.50–2.21)**

*Significant difference. *N*, number (of); *RR*, relative risk; *CI*, confidence interval; *ADR*, adverse drug reaction; *w/o*, without; *EPS*, extrapyramidal symptoms

#### Single and multiple imputations

Cutaneous reactions were the most common type of single-imputation ADD-associated ADR (203 cases), followed by neurological symptoms (excl. EPS; 191 cases) and liver dysfunction (114 cases). Among multiple imputation ADRs, neurological disorders were most common (150 cases), followed by delirium/confusion (118 cases) and metabolic disorders/electrolyte disturbances (111 cases).

Seven hundred twenty-four of 1263 ADRs in women (57.3% of all ADRs in women) imputed a single ADD, whereas this was the case in 346 of 616 ADRs in men (56.2% of all ADRs in men). The RR of ADRs imputing both a single ADD (f 0.478% vs m 0.375%; RR 1.27, CI 1.12–1.45) and multiple drugs (f 0.356% vs m 0.293%; RR 1.22, CI 1.05–1.41) was significantly higher in women than in men. The RR for neurological ADRs imputing a single ADD was higher for females (f 0.090% vs m 0.060%; RR 1.50, CI 1.10–2.06), especially serotonin syndrome/serotonergic ADRs (f 0.016% vs m 0.005%; RR 2.92, CI 1.11–7.66), which was not the case when considering “all cases” (i.e., single and multiple imputations together) or multiple imputations. Cutaneous reactions occurred with a higher RR both as single (f 0.109% vs m 0.041%; RR 2.64, CI 1.86–3.76) and multiple imputation ADRs in women (f 0.012% vs m 0.003%; RR 3.65, CI 1.08–12.40). Metabolic disorders imputing more than one drug had a significantly higher RR in women (f 0.061% vs m 0.020%; RR 3.14, CI 1.90–5.21)—this was primarily the case for hyponatremia (f 0.055% vs m 0.018%; RR 3.01, CI 1.79–5.06)—whereas the RR for metabolic ADRs imputing only one drug did not differ among sexes. Conversely, weight gain imputing a single ADD had a higher RR in women (f 0.026% vs m 0.009%; RR 3.04, CI 1.42–6.60), while weight gain imputing multiple drugs did not differ among sexes.

**Fig. 2:**
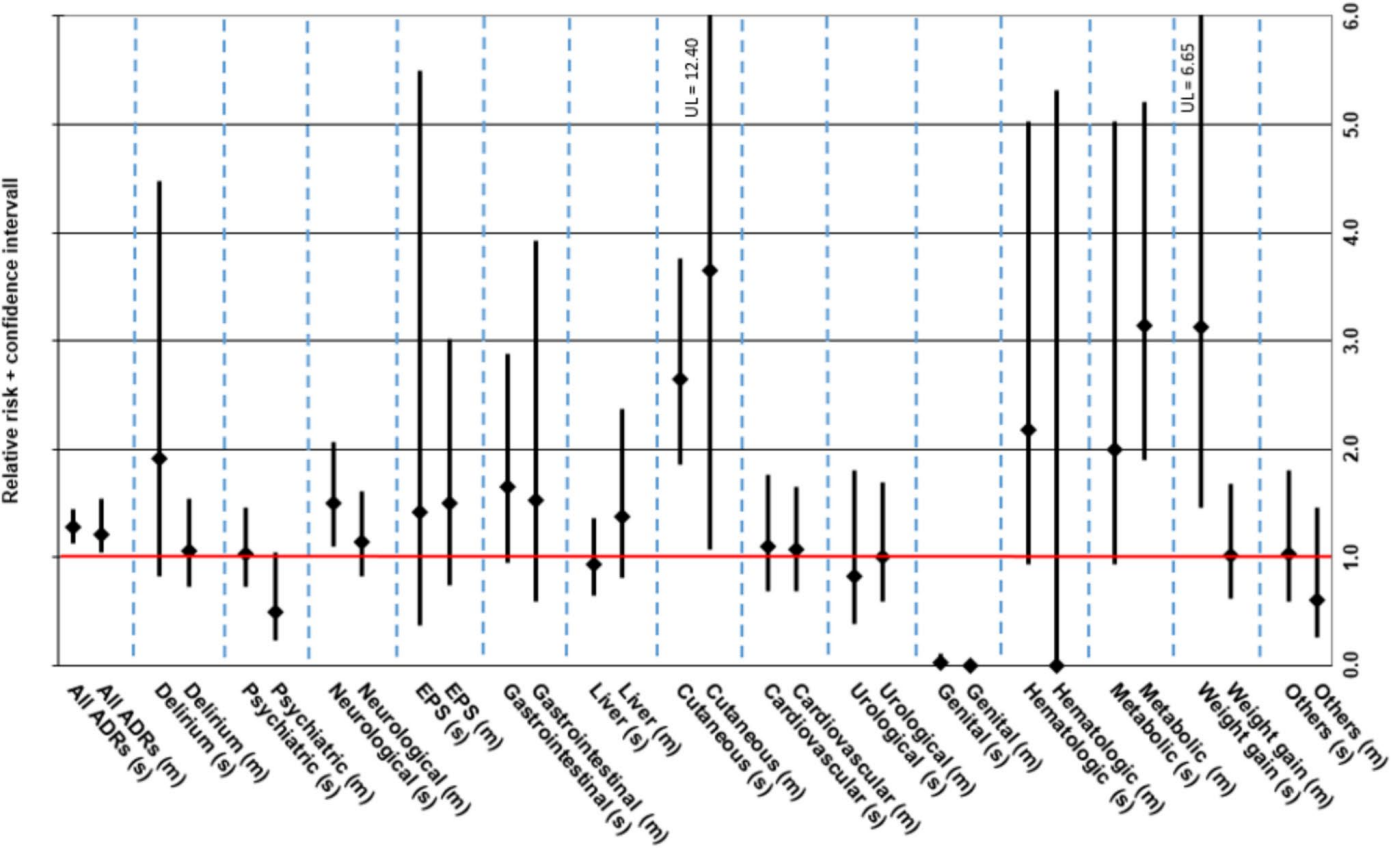
Relative risk (RR) incl. 95% confidence interval of single vs multiple imputation adverse drug reactions (ADRs) according to the affected organ system in females vs males. RR > 1 implies a higher ADR-risk for females compared to males; RR < 1 implies a lower ADR-risk for females compared to males. (s), single-imputation ADR; (m), multiple-imputation ADR; w/, with; w/o, without; EPS, extrapyramidal symptoms; UL, upper limit

The sex-specific RR of all ADRs as single and multiple imputations according to the affected organ system is depicted in Fig. 2. The exact number of ADR events and percent of patients affected, as well as the exact RRs including the 95% CIs of the affected organ system and of individual ADRs (cut-off ≥ 15 cases in all patients), can be found in the supplementary material (Suppl. Table 1).

### Adverse drug reactions associated with antidepressant drug classes and specific antidepressant drugs

#### All imputations (i.e., single and multiple imputation)

**Fig. 3:**
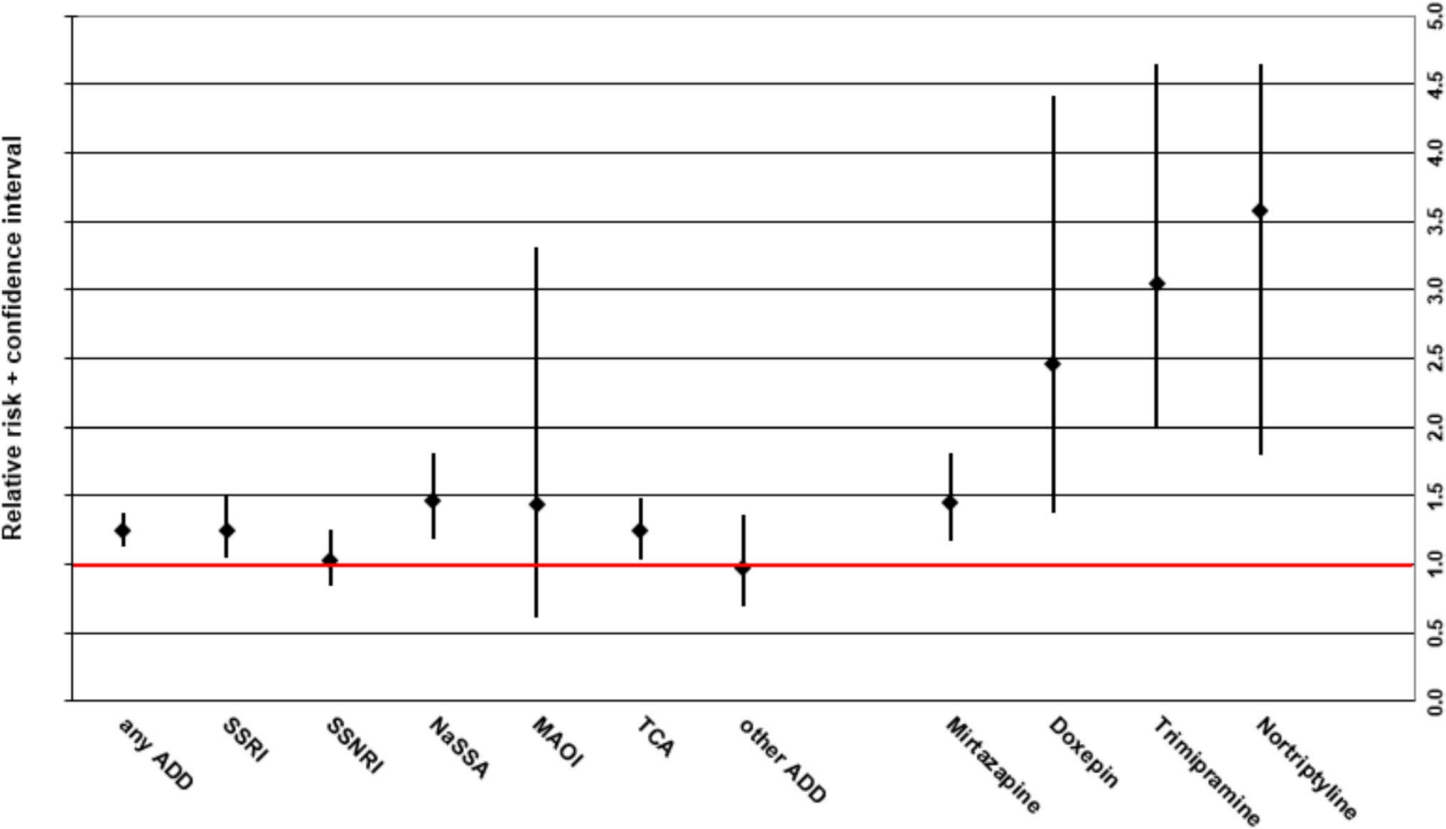
Relative risk (RR) incl. 95% confidence interval of drug reactions (ADRs; all imputations) for different antidepressant drug (ADD) groups and a selection of individual ADDs in females vs males for which we detected significant differences between sexes. The RR > 1 implies a higher ADR-risk for females compared to males; RR < 1 implies a lower ADR-risk for females compared to males. (s), single imputation ADR; (m), multiple imputation ADR; SSRI, selective serotonin reuptake inhibitor; SSNRI, selective serotonin–norepinephrine reuptake inhibitor; TCA, tricyclic antidepressant; NaSSA, noradrenergic and specific serotonergic antidepressant; MAOI, monoamine oxidase inhibitor; UL, upper limit

Women had a higher incidence of ADRs associated with SSRIs (f 0.61% vs m 0.49%; RR 1.25, CI 1.04–1.50), NaSSAs (f 0.71% vs m 0.48%; RR 1.46, CI 1.18–1.81), and TCAs (f 1.02% vs m 0.92%; RR 1.24, CI 1.03–1.49). The rate of ADRs did not differ among sexes for SSNRIs, MAOIs, or “other ADDs.” Among individual antidepressant drugs, sex-related differences were found only for mirtazapine (i.e., the most used NaSSA; m 0.68% vs m 0.47%; RR 1.46, CI 1.17–1.82), as well as the two TCAs doxepin (f 0.66% vs m 0.27%; RR 2.46, CI 1.37–4.41) and trimipramine (f 0.67% vs m 0.22%; RR 3.04, CI 1.99–4.65). None of the ADD classes or individual ADDs were associated with significantly higher RRs for ADRs among male patients (Fig. 3, Table 4).

**Table 4:** Incidence and relative risk of different types of adverse drug reactions (ADRs) associated with antidepressant drugs (all imputations) according to sex

Drug/drug group	Females			Males			RR (f vs m)
	***N* cases of ADRs**	***N* patients exposed to respective drug (group)**	**% of patients exposed to drug (group) with ADR**	***N* cases of ADRs**	***N* patients exposed to respective drug (group)**	**% of patients exposed to drug (group) with ADR**	**RR (95% CI)**
**Any antidepressant drug**	1263	151,426	**0.83%**	616	92,162	**0.67%**	1.25 (1.13–1.37)*
**SSRI**	342	55,926	**0.61%**	179	36,569	**0.49%**	1.25 (1.04–1.50)*
Citalopram	101	15,607	**0.65%**	49	9297	**0.53%**	**1.23 (0.87–1.73)**
Escitalopram	73	15,027	**0.49%**	41	10,640	**0.39%**	**1.26 (0.86–1.85)**
Sertraline	86	12,976	**0.66%**	45	8892	**0.51%**	**1.31 (0.91–1.88)**
Paroxetine	55	6349	**0.87%**	28	3949	**0.71%**	**1.22 (0.78–1.92)**
Fluoxetine	16	4132	**0.39%**	8	1716	**0.47%**	**0.83 (0.36–1.94)**
Fluvoxamine	11	1947	**0.56%**	9	2169	**0.41%**	**1.36 (0.57–3.28)**
**SSNRI**	273	36,521	**0.75%**	146	20,009	**0.73%**	**1.02 (0.84–1.25)**
Duloxetine	55	9591	**0.57%**	26	4752	**0.55%**	**1.05 (0.66–1.67)**
Venlafaxine	211	26,501	**0.80%**	118	15,058	**0.78%**	**1.02 (0.81–1.27)**
**NaSSA**	265	37,491	**0.71%**	124	25,691	**0.48%**	1.46 (1.18–1.81)*
Mirtazapine	243	35,727	**0.68%**	115	24,602	**0.47%**	1.46 (1.17–1.82)*
**MAOI**	35	3202	**1.09%**	12	1653	**0.73%**	**1.51 (0.78–2.89)**
**Tricyclic antidepressants**	366	35,767	**1.02%**	157	19,039	**0.82%**	1.24 (1.03–1.49)*
Amitriptyline	103	9345	**1.10%**	44	4744	**0.93%**	**1.19 (0.84–1.69)**
Doxepin	57	8606	**0.66%**	14	5205	**0.27%**	2.46 (1.37–4.41)*
Trimipramine	59	8852	**0.67%**	33	15,058	**0.22%**	3.04 (1.99–4.65)*
Clomipramine	66	3886	**1.70%**	33	2289	**1.44%**	**1.18 (0.78–1.78)**
**Other antidepressant drugs**	89	15,472	**0.58%**	55	9289	**0.59%**	**0.97 (0.69–1.36)**
Trazodone	31	8240	**0.38%**	21	4331	**0.48%**	**0.78 (0.45–1.35)**
Agomelatine	21	2805	**0.75%**	6	1343	**0.45%**	**1.68 (0.68–4.14)**
Bupropion	18	2249	**0.80%**	12	2273	**0.53%**	**1.52 (0.73–3.14)**
Reboxetine	14	1979	**0.71%**	16	1311	**1.22%**	**0.58 (0.28–1.18)**

*Significant difference. *N*, number (of); *f*, females; *m*, males; *RR*, relative risk; *CI*, confidence interval; *SSRI*, selective serotonin reuptake inhibitor; *SSNRI*, selective serotonin–norepinephrine reuptake inhibitor; *NaSSA*, noradrenergic and specific serotonergic antidepressant; *MAOI*, monoamine oxidase inhibitor

#### Single and multiple imputations

The RR for ADRs in NaSSA users (f 0.40% vs m 0.25%; RR 1.62, CI 1.25–2.33) and in TCA users (f 0.53% vs m 0.38%; RR 1.40, CI 1.07–21.83) was higher for women when considering ADRs implicating a single ADD. A significant sex-specific difference was not detected for SSRIs, SSNRIs, MAOIs, or “other ADDs.” However, several individual ADDs had a higher RR for ADRs imputing a single ADD but not for ADRs imputing multiple drugs in women than in men, including mirtazapine (f 0.39% vs m 0.23%; RR 1.71, CI 1.25–2.33) and doxepin (f 0.36% vs m 0.08%; RR 4.69, CI 1.66–13.27). The RR of ADRs imputing trimipramine was higher in females for both single- (f 0.32% vs m 0.09%; RR 3.55, CI 1.90–7.07) and multiple-imputation ADRs (f 0.35% vs m 0.13%; RR 2.64, CI 1.50–4.62).

**Fig. 4:**
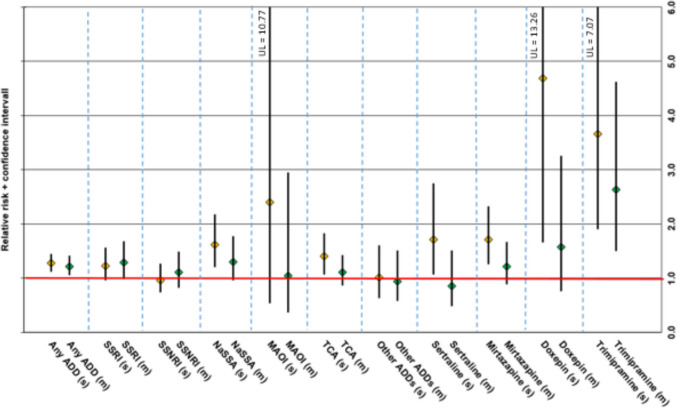
Relative risk (RR) incl. 95% confidence interval of single vs multiple imputation adverse drug reactions (ADRs) for different antidepressant drug (ADD) groups and a selection of individual ADDs in females vs males for which we detected significant differences between sexes. RR > 1 implies a higher ADR-risk for females compared to males; RR < 1 implies a lower ADR-risk for females compared to males. (s), single imputation ADR; (m), multiple imputation ADR; SSRI, selective serotonin reuptake inhibitor; SSNRI, selective serotonin–norepinephrine reuptake inhibitor; TCA, tricyclic antidepressant; NaSSA, noradrenergic and specific serotonergic antidepressant; MAOI, monoamine oxidase inhibitor; UL, upper limit

The sex-specific RR for ADRs implicating different ADD classes and a selection of individual ADDs for which differences were found as single and multiple imputations is shown in Fig. 4. The exact number of ADR events and percent of patients affected, as well as the exact RRs including the 95% CIs of the implicated ADDs (cut-off ≥ 15 cases in all patients), can be found in the supplementary material (single imputations, Suppl. Table 2; multiple imputations, Suppl. Table 3).

### Drug dosages in patients with and without adverse drug reactions according to sex

The median daily doses of the ADDs with the highest number of ADRs (cut-off ≥ 30 cases) are shown in Table 5. Among all patients exposed to the respective ADD, the median dose of most ADDs did not show relevant differences between men and women. Exceptions included duloxetine and escitalopram, which were both used at lower doses in women, whereas women treated with paroxetine received a higher median dosage than men. In patients with ADRs, there was mostly no difference in dosing between women and men except for amitriptyline and bupropion (both higher doses in men) and doxepin, paroxetine, and trimipramine (all dosed higher in women). When stratifying by patients with and without ADRs accordingly, women with bupropion-associated ADRs received a lower median dose than women without ADRs. In contrast, doxepin and maprotiline were both dosed higher in women with ADRs compared to those without ADRs. Among males, men with ADRs received higher median dosages of amitriptyline, clomipramine, and maprotiline and lower median dosages of escitalopram, reboxetine, and trimipramine.

**Table 5:** Median daily dosages in all patients exposed compared to patients with adverse drug reactions (ADRs) under treatment with imputed antidepressant drugs

Drug (cut of ≥ 30 ADRs total)	Females		Males	
	**Median (min/max) in mg/d**		**Median (min/max) in mg/d**	
	**All patients exposed**	**ADR cases**	**All patients exposed**	**ADR cases**
**Amitriptyline**	100 (10/350)	100 (25/375)	100 (10/300)	112.50 (25/400)
**Bupropion**	300 (150/600)	150 (75/300)	300 (37.5/600)	300 (75/300)
**Citalopram**	20 (10/250)	20 (10/100)	20 (5/100)	20 (10/80)
**Clomipramine**	125 (12.5/375)	150 (25/300)	125 (12.5/450)	150 (25/375)
**Doxepin**	100 (5/400)	125 (12.5/300)	100 (5/60)	100 (25/125)
**Duloxetine**	60 (15/240)	60 (30/120)	90 (20/360)	60 (30/180)
**Escitalopram**	10 (2.5/20)	10 (5/30)	15 (2/20)	10 (5/30)
**Maprotiline**	100 (10/300)	150 (30/250)	100 (12.5/300)	150 (50/250)
**Mirtazapine**	30 (3.75/30)	30 (7.5/90)	30 (15/30)	30 (7.5/90)
**Paroxetine**	30 (10/40)	30 (10/60)	20 (2/120)	20 (10/60)
**Reboxetine**	8 (1/16)	4 (2/14)	8 (2/12)	5 (1/12)
**Sertraline**	100 (10/400)	100 (25/250)	100 (10/150)	100 (15/250)
**Trazodone**	150 (10/600)	150 (25/350)	150 (10/600)	150 (50/450)
**Trimipramine**	100 (3/550)	100 (12.50/300)	100 (5/125)	75 (10/300)
**Venlafaxine**	150 (7.5/600)	150 (7.5/450)	150 (7.5/600)	150 (30/375)

*d*, day; *min.*, minimum; *max.*, maximum

Overall, dosage patterns were largely similar between individuals with and without ADRs with a few exceptions. In females with ADRs, the median dose of bupropion was lower than in all women treated with bupropion, whereas doxepin and maprotiline were used at higher dosages among those with ADRs. In men with ADRs, higher median doses of amitriptyline, clomipramine, and maprotiline were observed, whereas lower dosages were found for escitalopram, reboxetine, and trimipramine. Several extreme maximum dosages were found in the cohort of all patients exposed (e.g., citalopram, duloxetine, venlafaxine).

### Course, countermeasures, and risk factors of adverse drug reactions

A majority of both men (75.00%) and women (78.70%) with ADRs made a full recovery by the end of the observation period (i.e., generally when the patient was discharged from inpatient care) and received some kind of countermeasure to treat the ADR. Among the small group of patients with ADRs without countermeasures, men were significantly more likely not to have had any treatment for the ADR (m 3.73% vs f 1.27%; *p* < 0.001), while women were more likely to have received symptomatic treatment with other drugs (f 34.13% vs m 28.08%; *p* = 0.010). Regarding risk factors for ADRs, only pre-existing organ damage was more common among males (m 29.06% vs f 24.39%; *p* = 0.035; Table 6).

**Table 6:** Course, countermeasures, and risk factors of adverse drug reactions (ADRs) according to sex

	**Females (*N* = 1263)**		**Males (*N* = 616)**		***χ***^**2**^** test (f vs m)**	
	***N* cases**	**% of 1263 ADRs**	***N* cases**	**% of 616 ADRs**	***χ***^**2**^	***p*-value**
**Course of the ADR**						
Resulting in death	3	0.24%	5	0.81%	2.01	0.157
Full recovery be end of observation period	994	78.70%	462	75.00%	3.04	0.081
Improvement by end of observation period	174	13.78%	110	17.86%	5.06	0.025*
Unchanged by end of observation period	74	5.86%	34	5.52%	0.04	0.848
Permanent damage	5	0.40%	1	0.16%	0.17	0.684
**Countermeasures**^Ψ^						
None	16	1.27%	23	3.73%	11.21	< 0.001*
Reduction of dose	228	18.05%	111	18.02%	0	1.000
Discontinuation of drug	1052	83.29%	522	84.74%	0.54	0.464
Transfer to different ward/hospital	117	9.26%	58	9.42%	0	0.983
Symptomatic treatment with drugs	431	34.13%	173	28.08%	6.65	0.010*
Non-pharmacological treatment of symptoms	212	16.79%	114	18.51%	0.74	0.390
Diagnostic work-up	283	22.41%	142	23.05%	0.07	0.799
**Risk factors for ADRs**^Ψ^						
None	700	55.42%	335	54.38%	0.14	0.707
Risk factors present	563	44.58%	281	45.62%		
Susceptibility for ADRs	90	7.13%	40	6.49%	0.17	0.682
Pre-existing organ damage	308	24.39%	179	29.06%	4.47	0.035*

^Ψ^More than one item may apply. *Significant difference

In eight cases, the ADD-induced ADR resulted in death, which will be briefly described in the following (more details can be found in (Seifert et al. 2024)). Among women with fatal outcomes, one patient developed agranulocytosis under clozapine and mirtazapine, another woman suffered from a fatal seizure under venlafaxine, olanzapine, prednisolone, methotrexate, and fesoterodine, while another female suffered a bolus death under mianserin, olanzapine, lorazepam, and darifenacin. In males with fatal ADD-induced ADRs, two patients died of severe hypotension associated with the use of nortriptyline (one case) and tranylcypromine alongside several antihypertensive drugs (one case). A further patient died of gastrointestinal bleeding under citalopram and acetylsalicylic acid, while another patient developed pneumonia following severe sedation under mirtazapine, haloperidol, oxazepam, and prothipendyl. Finally, one male died of an ileus under amitriptyline, biperiden, pirenzepine, and five different antipsychotic drugs.

## Discussion

The present study analyzed severe ADRs occurring under treatment with ADDs and found that women have a significantly higher overall incidence of ADD-induced ADRs than men. While women were more likely to develop ADD-induced edema, allergic cutaneous reactions, and hyponatremia, men had a significantly higher incidence of sexual dysfunction. In general, the incidence of weight gain, however, did not differ between sexes. Certain ADDs including SSRIs, NaSSAs, and TCAs are associated with a higher incidence of ADRs when used in the treatment of women. Interestingly, no ADDs were identified as being associated with an increased incidence of ADRs in men.

One of the main questions that arises is why female sex is a risk factor for ADRs. Are women indeed more likely to experience ADRs, or are they simply more willing to communicate them? Aiming to better understand a patient’s experience of taking psychotropic drugs, Barbui et al. examined the subjective tolerability of psychotropic drugs, specifically antipsychotic drugs, and found that sex was the single strongest determinant, predicting a lower tolerance of ADRs (Barbui et al. 2005). Antipsychotic and antidepressant drugs are, of course, two distinct drug classes that have different pharmacological mechanisms; however, several of the most frequent ADRs, such as sedation, weight gain, and anticholinergic effects, are comparable. The lower tolerance of ADRs, reducing quality of life, may precipitate a woman’s tendency to report her discomfort. On the other hand, women’s potentially higher risk of ADRs can also be viewed in the broader context of gender inequality and discrimination in healthcare, treatment guidelines, and research. Structural biases, including the underrepresentation of women in clinical trials, male-dominant decision-making, and under-recognition of female-specific mental health needs, may contribute to insufficient treatment regimens for women (Hosang and Bhui 2018), thus putting them at risk for adverse health outcomes, such as ADRs.

When interpreting the findings of the present study, it is essential to bear in mind that polypharmacy predisposes for some types of ADRs (e.g., hyponatremia), but not for others (e.g., edema, allergic cutaneous reactions). Similarly, while some ADRs are dose-dependent (e.g., weight gain), others are not (e.g., edema, hyponatremia). This variability creates a complex interaction of potential risk factors, which affects the overall interpretation of ADR-risks. Our results suggest that the total number of drugs, as well as the number of psychotropic and antidepressant drugs (see Sect. “Average number of different drug groups”), did not differ between men and women in a clinically relevant manner, and neither did the dose of most ADDs. While the former finding is encouraging and in contrast to previous studies suggesting higher rates of polypharmacy in women with depression (Ghaed-Sharaf et al. 2022; Thunander Sundbom and Hedborg 2019), the latter is unfortunate. Under consideration of their respective metabolic rate, women in our sample tended to receive relatively higher doses. This may pose as a potential concern as previous research has suggested that women may require lower doses of certain types of ADDs. One reason for this is that women exhibit a higher plasma concentration–dose ratio of several ADDs than men (Haack et al. 2009). This particularly appears to affect several TCAs (e.g., amitriptyline, clomipramine, doxepin), but also mirtazapine and citalopram, which may put them at higher risk for dose-dependent ADRs (Unterecker et al. 2013; Hildebrandt et al. 2003). Further, the activity of cytochrome P450 (CYP) enzymes may differ among sexes, with men exhibiting a higher activity of CYP1A2 and 1E2, and women an increased metabolization via CYP3A4 (Aljohmani and Yildiz 2025; Trenaman et al. 2021). Although plasma concentrations were not routinely available for the present study, these effects may explain the higher propensity for ADRs in women we observed for mirtazapine, a substrate of CYP1A2. The median dose of mirtazapine (30 mg/day) did not differ between sexes or between patients with and without ADRs in this study. We also found a higher incidence of ADRs associated with doxepin in women. However, the median daily doses of doxepin were indeed highest in women with ADRs compared to women without ADRs and men with and without ADRs. Notably, we also found extreme maximum dosages for several ADDs including citalopram, duloxetine, and venlafaxine among all patients exposed but not among patients with ADRs. This finding indicates that these very high outlier doses were not primary contributors to ADRs, but rather appear to have been tolerated by the respective individuals, potentially reflecting personalized treatment strategies informed by therapeutic drug monitoring.

The willingness to tolerate certain types of ADRs may also be largely dependent on sex. Two of the ADD-related ADRs for which this discrepancy is most apparent are weight gain and sexual dysfunction. Women are more burdened by weight gain, whereas the opposite is the case for sexual dysfunction (Haack et al. 2009). It is, of course, important to acknowledge a patient’s subjective burden resulting from an ADR, but it does not imply that either ADD-induced weight gain or sexual dysfunction is indeed more common in men or women. In fact, sexual dysfunction associated with ADD use appears to affect both sexes equally, but men report it more frequently (Montejo et al. 2019). The results from our study demonstrate this tendency and challenge the notion that women are more likely to report ADRs, as the detection of sexual dysfunction relies on a patient’s own report. The present study indicates an 18-fold higher incidence for sexual dysfunction in men. ADD-induced sexual dysfunction is generally attributed to serotonergic effects (Rothmore 2020; Montejo et al. 2019), indicating that ADD with pronounced serotonergic properties—and specifically inhibition of the serotonin transporter—like SSRIs and SSNRIs, are of particular concern (Serretti and Chiesa 2009). Bupropion and mirtazapine, on the other hand, appear to be more favorable choices. A previous AMSP study examining drug utilization trends in patients with major depressive disorder identified a higher use of both mirtazapine and bupropion and lower use of SSRIs among men in comparison to women (Seifert et al. 2021b), perhaps in an attempt to bypass sexual dysfunction.

Weight gain, unlike sexual dysfunction, is not merely reliant on a patient’s own account and is easily objectified. While the present study did detect a slightly higher susceptibility for changes in weight in females, this did not reach a level of significance for ADD-induced weight gain. This finding suggests that it affects men and women equally, at least when considering all events of this ADR as a whole group. Interestingly, we found that women were more likely to experience weight gain as a result of a single ADD, whereas the incidence of weight gain implicating more than one drug did not differ between sexes. When considering both single and multiple imputations, the risk of weight gain also did not show sex differences. In general, all ADDs apart from agomelatine and bupropion have been associated with clinically relevant weight gain, with some drugs (e.g., mirtazapine, paroxetine, TCAs including amitriptyline and trimipramine) exhibiting a higher risk than others (e.g., fluoxetine, venlafaxine) (Gill et al. 2020). Unfortunately, due to limited case numbers, the present study is unable to disclose whether a certain ADD coincides with any sex-specific trends in this regard; however, several ADDs with a high risk for weight gain, including mirtazapine and trimipramine, did present with an increased incidence of this ADR in women.

Another interesting finding in the present study was that women were more likely to experience serotonin syndrome or other serotonergic ADRs related to a single ADD. This was not the case for serotonergic ADRs implicating more than one drug. Serotonergic ADRs appear to be dose-related (Scotton et al. 2019), and as discussed above, women may have higher plasma concentrations of ADDs than men (Bigos et al. 2009), predisposing them for this type of ADR. However, as serotonergic ADRs—and serotonin syndrome in particular—are most commonly precipitated by the simultaneous use of multiple drugs with serotonergic properties (Scotton et al. 2019), the incidence of this ADR subtype implicating multiple drugs did not differ among sexes.

Increasing evidence implies that women are generally more susceptible to idiosyncratic ADRs (i.e., ADRs unrelated to dose) such as allergic reactions (Haack et al. 2009), perhaps due to differences in immunological response which also predisposes women to an increased prevalence of autoimmune diseases such as systemic lupus erythematodes (Rademaker 2001). Unfortunately, as these ADRs are largely unrelated to dose and occur unpredictably, they are nearly impossible to avoid. Drug-induced edema, for which the exact mechanisms are not completely understood but may involve both allergic reactions (Ng et al. 2003) as well as vasodilatatory properties of certain drugs, is also significantly more common among women (Joseph et al. 2023). In fact, it was the single ADR with the highest risk increase in females in the present study. A previous study examining psychotropic drug-induced edema using AMSP data found that mirtazapine was the ADD with the highest incidence of this ADR (Engel et al. 2024). This may well explain the higher ADR incidence in female mirtazapine-users that this study detected, especially considering the lack of sex disparity for weight gain. Also generally recognized as idiosyncratic in nature, we found a higher incidence of ADD-induced hematologic ADRs in women in general. However, the individual reactions, including the most common type (i.e., abnormal bleeding), did not show a sex preference probably due to low case numbers. A previous study reported SSRI-related hematological reactions—mainly resulting in different types of abnormal bleeding—to occur almost exclusively in women (Spigset 1999).

We found a significantly higher incidence of severe hyponatremia in women, in particular as an ADR imputing more than one drug. A previous study analyzing AMSP data elucidated that among ADDs, SSRIs and SSNRIs carry the greatest potential for inducing this ADR, especially when used in combination with other drugs associated with hyponatremia (Seifert et al. 2021c). The same study found that female SSNRI-users aged ≥ 65 years concomitantly treated with other hyponatremia-inducing drugs are the most at-risk patient subgroup (Seifert et al. 2021c). While we found a higher ADR incidence in female SSRI-users, the incidence of SSNRI-associated ADRs did not differ among sexes. In general, the ADR-risk for SSNRIs was higher than that of SSRIs, perhaps suggesting that SSNRI’s additional noradrenergic properties may predispose both sexes to ADRs.

Finally, symptomatic hyperprolactinemia and galactorrhea were more common among women in the present study, a largely unsurprising finding. While antipsychotic drugs with strong antagonist properties at dopamine D2 receptors are well known for this ADR (Peuskens et al. 2014), ADDs have also been associated with increasing prolactin. Prolactin-elevating effects have been reported for nearly all ADDs—with the exception of mirtazapine—and are most frequently seen under serotonergic ADDs. Compared to antipsychotic drug-induced events, significant elevations in prolactin levels caused by ADDs are considered rare; however, if they do occur, women appear to be more susceptible (Coker and Taylor 2010; Madhusoodanan et al. 2010).

## Strengths and limitations

As a pharmacovigilance program, AMSP offers complementary insights to data derived from clinical trials. Unlike clinical trials with strict inclusion criteria, this pharmacovigilance program is able to monitor patients under “real-life” circumstances, such as those with severe mental illnesses, somatic comorbidities, and patients with polypharmacy. AMSP is further characterized not only by a standardized and established methodology for the assessment of ADRs in routine inpatient care in psychiatry, but also by a rigorous multi-level expert review process, ensuring a high degree of accuracy in the causality assessment of drugs involved in reported ADRs.

Despite these strengths, pharmacovigilance programs such as AMSP are confronted with several limitations, one of which is its observational nature, rather than a randomized controlled clinical trial. This may limit the reliability of evidence. Prior AMSP studies have demonstrated that drug utilization trends have changed over time (Seifert et al. 2021a; Toto et al. 2019). Regional drug use patterns and drug availability also vary in the participating countries of Germany, Switzerland (participation since 1993), and Austria (since 2001). Moreover, the structure and type of information stored in the AMSP database are limited. For example, it is not possible to determine if individual patients developed multiple ADRs. Apart from basic demographic information and a detailed report on drug use, the database does not include any significant clinical parameters such as treatment response or non-pharmacological treatments. The present study includes psychiatric inpatients, a population which may not be representative of most patients treated with ADDs within the outpatient setting.

Underreporting of ADRs also remains a concern, as documentation of ADRs is generally not part of the physicians’ routine clinical work. Consequently, reporting may vary depending on personal time, motivation, but also institutional resources. The possibility of individual and systemic bias must also be acknowledged, which may contribute to a higher reporting and detection of ADRs occurring in drugs with well-known ADR-related concerns (e.g., anticholinergic ADRs under TCAs), ADRs that are more easily objectified (e.g., low serum sodium concentration in hyponatremia), or in newly available drugs.

## Conclusion and clinical implications

The overall rate of severe ADRs under treatment with ADDs is low. Women have a significantly higher risk of being affected by ADD-induced ADRs, especially hyponatremia, edema, and allergic reactions, in comparison to men. Sexual dysfunction was the only ADR more common in men. Certain ADD classes, including SSRIs, NaSSAs, and TCAs, are more likely to cause ADRs in women than in men. These findings emphasize the relevance of sex-specific pharmacovigilance in psychiatry. An in-depth understanding of these differences in drug safety is essential in providing patients with individualized treatment options, informed drug selection, and improved tolerability. Incorporating routine ADR monitoring into clinical practice enables early detection or ideally prevention of harmful and burdensome ADRs, thereby promoting adherence and improving treatment outcomes.

## Supplementary Information

Below is the link to the electronic supplementary material.

ESM1 (DOCX 31.0 KB)

## Data Availability

Data on an individual level generated and/or analysed during the current study are not publicly available due to data protection regulations and ethical considerations. Summarized data on the prevalence of ADRs and drug use relevant to the present study is provided in the tables and supplementary material of this manuscript.

## Abbreviations

ADD: Antidepressant drug
ADR: Adverse drug reaction
AMSP: Drug Safety in Psychiatry (German: “Arzneimittelsicherheit in der Psychiatrie”)
CI: Confidence interval
d: Day
df: Degrees of freedom
EPS: Extrapyramidal symptoms
f: Female
ICD-10: International Classification of Disease, 10th Version
m: Male
MAOI: Monoamine oxidase inhibitor
Max.: Maximum
Min.: Minimum
N: Number (of)
NaSSA: Noradrenergic and specific serotonergic antidepressant
SSNRI: Selective serotonin-norepinephrine reuptake inhibitor
SSRI: Selective serotonin reuptake inhibitor
TCA: Tricyclic antidepressant drug
w/: With
w/o: Without
