# Supplementary material for: A 24-year pharmacovigilance study on sex differences in adverse drug reactions to antidepressant drugs
Source: Naunyn Schmiedebergs Arch Pharmacol. 2025 Dec 13;399(5):7597–613. doi: 10.1007/s00210-025-04900-7 (PMC13053531; doi:10.1007/s00210-025-04900-7)
Supplement: Supplementary file 1 — (DOCX 31.0 KB) [file 210_2025_4900_MOESM1_ESM.docx]

**Suppl. Table 1:** Incidence and relative risk of different types of adverse drug reactions (ADRs; single imputations) according to sex

| **Adverse drug reaction (cut-off 15 ADRs total)** | **Females (N=151,426)** | | **Males (N=92,162)** | |  |
| --- | --- | --- | --- | --- | --- |
|  | **All events of the ADR** | | **All events of the ADR** | |  |
|  | **N cases** |  | **N cases** |  |  |
|  |  | **% of females** |  | **% of males** | **RR (95% CI)** |
|  |  |  |  |  |  |
| **All ADRs** | 724 | **0.478%** | 346 | **0.375%** | **1.27 (1.12–1.45)*** |
| **Delirium, confusion** | 22 | **0.015%** | 7 | **0.008%** | **1.91 (0.82–4.48)** |
| **Psychiatric symptoms w/o delirium** | 88 | **0.058%** | 52 | **0.056%** | **1.03 (0.73–1.45)** |
| Psychosis/(pseudo-) hallucinations | 11 | **0.007%** | 6 | **0.007%** | **1.12 (0.41–3.02)** |
| Restlessness/agitation | 29 | **0.019%** | 18 | **0.020%** | **0.98 (0.54–1.77)** |
| Sedation | 7 | **0.005%** | 4 | **0.004%** | **1.07 (0.31–3.64)** |
| Suicidality | 11 | **0.007%** | 8 | **0.009%** | **0.84 (0.34–2.08)** |
| Nightmares | 16 | **0.011%** | 6 | **0.007%** | **1.62 (0.64–4.15)** |
| **Neurological symptoms w/o EPS** | 136 | **0.090%** | 55 | **0.060%** | **1.50 (1.10–2.06)*** |
| Seizures | 12 | **0.008%** | 3 | **0.003%** | **2.43 (0.69–8.63)** |
| Myoclonus | 5 | **0.003%** | 2 | **0.002%** | **1.52 (0.30–7.84)** |
| Ataxia | 2 | **0.001%** | 3 | **0.003%** | **0.41 (0.07–2.43)** |
| Tremor | 13 | **0.009%** | 4 | **0.004%** | **1.98 (0.64–6.07)** |
| Vision disorders | 10 | **0.007%** | 2 | **0.002%** | **3.04 (0.67–13.89)** |
| Serotonin syndrome, serotonergic ADRs | 24 | **0.016%** | 5 | **0.005%** | **2.92 (1.11–7.66)*** |
| Restless legs/arms | 28 | **0.018%** | 18 | **0.020%** | **0.95 (0.52–1.71)** |
| Vertigo | 4 | **0.003%** | 6 | **0.007%** | **0.41 (0.11–1.44)** |
| Speech disorders | 2 | **0.001%** | 6 | **0.007%** | **0.20 (0.04–1.01)** |
| **EPS** | 7 | **0.005%** | 3 | **0.003%** | **1.42 (0.37–5.49)** |
| **Gastrointestinal disorders** | 46 | **0.030%** | 17 | **0.018%** | **1.65 (0.94–2.87)** |
| (Sub)ileus/severe constipation | 6 | **0.004%** | 2 | **0.002%** | **1.83 (0.37–9.05)** |
| Nausea/vomiting | 20 | **0.013%** | 5 | **0.005%** | **2.43 (0.91–6.49)** |
| Diarrhea | 13 | **0.009%** | 7 | **0.008%** | **1.13 (0.45–2.83)** |
| **Liver dysfunction** | 69 | **0.046%** | 45 | **0.049%** | **0.93 (0.64–1.36)** |
| **Cutaneous reactions** | 165 | **0.109%** | 38 | **0.041%** | **2.64 (1.86–3.76)*** |
| Edema | 67 | **0.044%** | 5 | **0.005%** | **8.16 (3.29–20.23)*** |
| Allergic cutaneous reactions | 87 | **0.057%** | 30 | **0.033%** | **1.77 (1.17–2.67)*** |
| **Cardiovascular disorders** | 49 | **0.032%** | 27 | **0.029%** | **1.10 (0.69–1.77)** |
| (Orthostatic) syncope | 11 | **0.007%** | 4 | **0.004%** | **1.67 (0.53–5.26)** |
| Symptomatic hypotension w/ vertigo | 6 | **0.004%** | 2 | **0.002%** | **1.83 (0.37–9.05)** |
| Hypertension | 15 | **0.010%** | 7 | **0.008%** | **1.30 (0.53–3.20)** |
| Arrhythmia | 12 | **0.008%** | 12 | **0.013%** | **0.61 (0.27–1.35)** |
| **Urological dysfunction** | 15 | **0.010%** | 11 | **0.012%** | **0.83 (0.38–1.81)** |
| Urinary retention | 14 | **0.009%** | 8 | **0.009%** | **1.07 (0.45–2.54)** |
| **Sexual dysfunction** | 2 | **0.001%** | 49 | **0.053%** | **0.02 (0.01–0.10)*** |
| Erectile dysfunction, impotence | 0 | **0.000%** | 33 | **0.036%** | **–** |
| **Hematologic disorders** | 25 | **0.017%** | 7 | **0.008%** | **2.17 (0.94–5.03)** |
| Neutropenia | 7 | **0.005%** | 3 | **0.003%** | **1.42 (0.37–5.49)** |
| Abnormal bleeding | 11 | **0.007%** | 1 | **0.001%** | **6.69 (0.86–51.86)** |
| **Metabolic disorders, electrolyte imbalances** | 23 | **0.015%** | 7 | **0.008%** | **2.00 (0.86–4.66)** |
| Hyponatremia* | 18 | **0.012%** | 5 | **0.005%** | **2.19 (0.81–5.90)** |
| Symptomatic hyperprolactinemia/galactorrhea* | 5 | **0.003%** | 0 | **0.000%** | **–** |
| **Weight gain** | 41 | **0.027%** | 8 | **0.009%** | **3.12 (1.46–6.65)*** |
| **Others** | 34 | **0.022%** | 20 | **0.022%** | **1.03 (0.60–1.80)** |
| Falls | 1 | **0.001%** | 0 | **0.000%** | **–** |
| Hyperhidrosis | 18 | **0.012%** | 9 | **0.010%** | **1.22 (0.55–2.71)** |

*indicates a significant result

N: number (of); RR: relative risk; CI: confidence interval; ADR: adverse drug reaction; w/o: without; EPS: extrapyramidal symptoms

**Suppl. Table 2**: Incidence and relative risk of different types of adverse drug reactions (ADRs; multiple imputations) according to sex

| **Adverse drug reaction (cut-off 15 ADRs total)** | **Females (N=151,426)** | | **Males (N=92,162)** | |  |
| --- | --- | --- | --- | --- | --- |
|  | **All events of the ADR** | | **All events of the ADR** | |  |
|  | **N cases** |  | **N cases** |  |  |
|  |  | **% of females** |  | **% of males** | **RR (95% CI)** |
|  |  |  |  |  |  |
| **All ADRs** | 539 | **0.356%** | 270 | **0.293%** | **1.22 (1.05–1.41)*** |
| **Delirium, confusion** | 75 | **0.050%** | 43 | **0.047%** | **1.06 (0.73–1.54)** |
| **Psychiatric symptoms w/o delirium** | 12 | **0.008%** | 15 | **0.016%** | **0.49 (0.23–1.04)** |
| Psychosis/(pseudo-) hallucinations | 3 | **0.002%** | 2 | **0.002%** | **0.91 (0.15–5.46)** |
| Restlessness/agitation | 4 | **0.003%** | 8 | **0.009%** | **0.30 (0.09–1.01)** |
| Sedation | 2 | **0.001%** | 2 | **0.002%** | **0.61 (0.09–4.32)** |
| Suicidality | 0 | **0.000%** | 1 | **0.001%** | **–** |
| Nightmares | 0 | **0.000%** | 1 | **0.001%** | **–** |
| **Neurological symptoms w/o EPS** | 98 | **0.065%** | 52 | **0.056%** | **1.15 (0.82–1.61)** |
| Seizures | 31 | **0.020%** | 22 | **0.024%** | **0.86 (0.50–1.48)** |
| Myoclonus | 11 | **0.007%** | 3 | **0.003%** | **2.23 (0.62–8.00)** |
| Ataxia | 8 | **0.005%** | 2 | **0.002%** | **2.43 (0.52–11.46)** |
| Tremor | 16 | **0.011%** | 4 | **0.004%** | **2.43 (0.81–7.28)** |
| Vision disorders | 2 | **0.001%** | 1 | **0.001%** | **1.22 (0.11–13.42)** |
| Serotonin syndrome, serotonergic ADRs | 12 | **0.008%** | 11 | **0.012%** | **0.66 (0.29–1.50)** |
| Restless legs/arms | 2 | **0.001%** | 1 | **0.001%** | **1.22 (0.11–13.42)** |
| Headache/migraine | 8 | **0.005%** | 3 | **0.003%** | **–** |
| Vertigo | 3 | **0.002%** | 3 | **0.003%** | **0.61 (0.12–3.02)** |
| Speech disorders | 5 | **0.003%** | 4 | **0.004%** | **0.76 (0.20–2.83)** |
| **EPS** | 27 | **0.018%** | 11 | **0.012%** | **1.49 (0.74–3.01)** |
| **Gastrointestinal disorders** | 15 | **0.010%** | 6 | **0.007%** | **1.52 (0.59–3.92)** |
| (Sub)ileus/severe constipation | 10 | **0.007%** | 5 | **0.005%** | **1.22 (0.42–3.56)** |
| Nausea/vomiting | 1 | **0.001%** | 1 | **0.001%** | **0.61 (0.04–9.73)** |
| Diarrhea | 1 | **0.001%** | 0 | **0.000%** | **–** |
| **Liver dysfunction** | 43 | **0.028%** | 19 | **0.021%** | **1.38 (0.80–2.36)** |
| **Cutaneous reactions** | 18 | **0.012%** | 3 | **0.003%** | **3.65 (1.08–12.40)*** |
| Edema | 16 | **0.011%** | 3 | **0.003%** | **3.25 (0.95–11.14)** |
| Allergic cutaneous reactions | 0 | **0.000%** | 1 | **0.001%** | **–** |
| **Cardiovascular disorders** | 56 | **0.037%** | 32 | **0.035%** | **1.07 (0.69–1.64)** |
| (Orthostatic) syncope | 32 | **0.021%** | 19 | **0.021%** | **1.03 (0.58–1.81)** |
| Symptomatic hypotension w/ vertigo | 7 | **0.005%** | 6 | **0.007%** | **0.71 (0.24–2.11)** |
| Hypertension | 0 | **0.000%** | 0 | **0.000%** | **–** |
| Arrhythmia | 13 | **0.009%** | 5 | **0.005%** | **1.58 (0.56–4.44)** |
| **Urological dysfunction** | 36 | **0.024%** | 22 | **0.024%** | **1.00 (0.59–1.69)** |
| Urinary retention | 34 | **0.022%** | 21 | **0.023%** | **0.99 (0.57–1.70)** |
| **Sexual dysfunction*** | 0 | **0.000%** | 10 | **0.011%** | **–** |
| Erectile dysfunction, impotence | 0 | **0.000%** | 8 | **0.009%** | **–** |
| **Hematologic disorders** | 16 | **0.011%** | 5 | **0.005%** | **1.95 (0.71–5.32)** |
| Neutropenia | 5 | **0.003%** | 1 | **0.001%** | **3.04 (0.36–26.05)** |
| Abnormal bleeding | 5 | **0.003%** | 3 | **0.003%** | **1.01 (0.24–4.24)** |
| **Metabolic disorders, electrolyte imbalances** | 93 | **0.061%** | 18 | **0.020%** | **3.14 (1.90–5.21)*** |
| Hyponatremia | 84 | **0.055%** | 17 | **0.018%** | **3.01 (1.79–5.06)*** |
| Symptomatic hyperprolactinemia /galactorrhea | 9 | **0.006%** | 1 | **0.001%** | **5.48 (0.69–43.24)** |
| **Weight gain** | 39 | **0.026%** | 24 | **0.026%** | **1.01 (0.61–1.68)** |
| **Others** | 10 | **0.007%** | 10 | **0.011%** | **0.61 (0.25–1.46)** |
| Falls | 8 | **0.005%** | 6 | **0.007%** | **0.81 (0.28–2.34)** |
| Hyperhidrosis | 1 | **0.001%** | 2 | **0.002%** | **0.30 (0.03–3.36)** |

*indicates a significant result

N: number (of); RR: relative risk; CI: confidence interval; ADR: adverse drug reaction; w/o: without; EPS: extrapyramidal symptoms

**Suppl. Table 3**: Incidence and relative risk of different types of adverse drug reactions (ADRs) associated with antidepressant drugs (single imputations) according to sex

| **Drug/drug group** |  | **Females** |  |  | **Males** |  | **RR (f vs. m)** |
| --- | --- | --- | --- | --- | --- | --- | --- |
|  | **N cases of ADRs** | **N patients exposed to respective drug (group)** | **% of patients exposed to drug (group) with ADR** | **N cases of ADRs** | **N patients exposed to respective drug (group)** | **% of patients exposed to drug (group) with ADR** | **RR (95%)** |
| **Any antidepressant drug** | 724 | 151,426 | **0.48%** | 346 | 92,162 | **0.38%** | **1.27 (1.12–1.45)*** |
| **SSRI** | 185 | 55,926 | **0.33%** | 99 | 36,569 | **0.27%** | **1.22 (0.96–1.56)** |
| Citalopram | 48 | 15,607 | **0.31%** | 28 | 9297 | **0.30%** | **1.02 (0.64–1.63)** |
| Escitalopram | 34 | 15,027 | **0.23%** | 25 | 10,640 | **0.23%** | **0.96 (0.57–1.61)** |
| Sertraline | 60 | 12,976 | **0.46%** | 24 | 8892 | **0.27%** | **1.71 (1.07–2.75)*** |
| Paroxetine | 30 | 6349 | **0.47%** | 13 | 3949 | **0.33%** | **1.44 (0.75–2.75)** |
| Fluoxetine | 5 | 4132 | **0.12%** | 4 | 1716 | **0.23%** | **0.52 (0.14–1.93)** |
| Fluvoxamine | 8 | 1947 | **0.41%** | 5 | 2169 | **0.23%** | **1.78 (0.58–5.44)** |
| **SSNRI** | 142 | 36,521 | **0.39%** | 81 | 20,009 | **0.40%** | **0.96 (0.73–1.26)** |
| Duloxetine | 33 | 9591 | **0.34%** | 15 | 4752 | **0.32%** | **1.09 (0.59–2.00)** |
| Venlafaxine | 106 | 26,501 | **0.40%** | 65 | 15,058 | **0.43%** | **0.93 (0.68–1.26)** |
| **NaSSA** | 149 | 37,491 | **0.40%** | 63 | 25,691 | **0.25%** | **1.62 (1.21–2.17)*** |
| Mirtazapine | 139 | 35,727 | **0.39%** | 56 | 24,602 | **0.23%** | **1.71 (1.25–2.33)*** |
| **MAOI** | 17 | 3202 | **0.53%** | 6 | 1653 | **0.36%** | **1.46 (0.58–3.70)** |
| **Tricyclic antidepressants** | 189 | 35,767 | **0.53%** | 72 | 19,039 | **0.38%** | **1.40 (1.07–1.83)*** |
| Amitriptyline | 54 | 9345 | **0.58%** | 18 | 4744 | **0.38%** | **1.52 (0.89–2.59)** |
| Doxepin | 31 | 8606 | **0.36%** | 4 | 5205 | **0.08%** | **4.69 (1.66–13.27)*** |
| Trimipramine | 28 | 8852 | **0.32%** | 13 | 15,058 | **0.09%** | **3.66 (1.90–7.07)*** |
| Clomipramine | 25 | 3886 | **0.64%** | 17 | 2289 | **0.74%** | **0.87 (0.47–1.60)** |
| Imipramine | 2 | 747 | **0.27%** | 2 | 437 | **0.46%** | **0.59 (0.08–4.14)** |
| Maprotiline | 20 | 2193 | **0.91%** | 8 | 946 | **0.85%** | **1.08 (0.48–2.44)** |
| **Other antidepressant drugs** | 47 | 15,472 | **0.30%** | 28 | 9289 | **0.30%** | **1.01 (0.63–1.61)** |
| Trazodone | 8 | 8240 | **0.10%** | 7 | 4331 | **0.16%** | **0.60 (0.22–1.66)** |
| Agomelatine | 15 | 2805 | **0.53%** | 4 | 1343 | **0.30%** | **1.80 (0.60–5.40)** |
| Bupropion | 13 | 2249 | **0.58%** | 5 | 2273 | **0.22%** | **2.63 (0.94–7.36)** |
| Reboxetine | 7 | 1979 | **0.35%** | 10 | 1311 | **0.76%** | **0.46 (0.18–1.22)** |

*indicates a significant result

N: number (of); f: females; m: males; RR: relative risk; CI: confidence interval; SSRI: selective serotonin reuptake inhibitor; SSNRI: selective serotonin-norepinephrine reuptake inhibitor; NaSSA: noradrenergic and specific serotonergic antidepressant; MAOI: monoamine oxidase inhibitor

**Suppl. Table 4**: Incidence and relative risk of different types of adverse drug reactions (ADRs) associated with antidepressant drugs (multiple imputations) according to sex

| **Drug/drug group** |  | **Females** |  |  | **Males** |  | **RR (f vs. m)** |
| --- | --- | --- | --- | --- | --- | --- | --- |
|  | **N cases of ADRs** | **N patients exposed to respective drug (group)** | **% of patients exposed to drug (group) with ADR** | **N cases of ADRs** | **N patients exposed to respective drug (group)** | **% of patients exposed to drug (group) with ADR** | **RR (95%)** |
| **Any antidepressant drug** | 534 | 151,426 | **0.35%** | 267 | 92,162 | **0.29%** | **1.22 (1.05–1.41)*** |
| **SSRI** | 157 | 55,926 | **0.28%** | 80 | 36,569 | **0.22%** | **1.28 (0.98–1.68)** |
| Citalopram | 53 | 15,607 | **0.34%** | 21 | 9297 | **0.23%** | **1.50 (0.91–2.49)** |
| Escitalopram | 39 | 15,027 | **0.26%** | 16 | 10,640 | **0.15%** | **1.73 (0.97–3.09)** |
| Sertraline | 26 | 12,976 | **0.20%** | 21 | 8892 | **0.24%** | **0.85 (0.48–1.51)** |
| Paroxetine | 25 | 6349 | **0.39%** | 15 | 3949 | **0.38%** | **1.04 (0.55–1.96)** |
| Fluoxetine | 11 | 4132 | **0.27%** | 4 | 1716 | **0.23%** | **1.14 (0.36–3.58)** |
| Fluvoxamine | 3 | 1947 | **0.15%** | 4 | 2169 | **0.18%** | **0.84 (0.19–3.73)** |
| **SSNRI** | 131 | 36,521 | **0.36%** | 65 | 20,009 | **0.32%** | **1.10 (0.82–1.49)** |
| Duloxetine | 22 | 9591 | **0.23%** | 11 | 4752 | **0.23%** | **0.99 (0.48–2.04)** |
| Venlafaxine | 105 | 26,501 | **0.40%** | 53 | 15,058 | **0.35%** | **1.13 (0.81–1.57)** |
| **NaSSA** | 116 | 37,491 | **0.31%** | 61 | 25,691 | **0.24%** | **1.30 (0.96–1.78)** |
| Mirtazapine | 104 | 35,727 | **0.29%** | 59 | 24,602 | **0.24%** | **1.21 (0.88–1.67)** |
| **MAOI** | 18 | 3202 | **0.56%** | 6 | 1653 | **0.36%** | **1.55 (0.62–3.89)** |
| **Tricyclic antidepressants** | 177 | 35,767 | **0.49%** | 85 | 19,039 | **0.45%** | **1.11 (0.86–1.43)** |
| Amitriptyline | 49 | 9345 | **0.52%** | 26 | 4744 | **0.55%** | **0.96 (0.60–1.54)** |
| Doxepin | 26 | 8606 | **0.30%** | 10 | 5205 | **0.19%** | **1.57 (0.76–3.26)** |
| Trimipramine | 31 | 8852 | **0.35%** | 20 | 15,058 | **0.13%** | **2.64 (1.50–4.62)*** |
| Clomipramine | 41 | 3886 | **1.06%** | 16 | 2289 | **0.70%** | **1.51 (0.85–2.68)** |
| **Other antidepressant drugs** | 42 | 15,472 | **0.27%** | 27 | 9289 | **0.29%** | **0.93 (0.58–1.51)** |
| Trazodone | 23 | 8240 | **0.28%** | 14 | 4331 | **0.32%** | **0.86 (0.44–1.68)** |
| Agomelatine | 6 | 2805 | **0.21%** | 2 | 1343 | **0.15%** | **1.44 (0.29–7.11)** |
| Bupropion | 5 | 2249 | **0.22%** | 7 | 2273 | **0.31%** | **0.72 (0.23–2.27)** |
| Reboxetine | 7 | 1979 | **0.35%** | 6 | 1311 | **0.46%** | **0.77 (0.26–2.29)** |

*indicates a significant result

N: number (of); f: females; m: males; RR: relative risk; CI: confidence interval; SSRI: selective serotonin reuptake inhibitor; SSNRI: selective serotonin-norepinephrine reuptake inhibitor; NaSSA: noradrenergic and specific serotonergic antidepressant; MAOI: monoamine oxidase inhibitor
